# Influence of Water, NaCl and Citric Acid Soaking Pre-Treatments on Acrylamide Content in French Fries Prepared in Domestic Conditions

**DOI:** 10.3390/foods11091204

**Published:** 2022-04-21

**Authors:** Mioara Negoiță, Adriana Laura Mihai, Gabriela Andreea Horneț

**Affiliations:** National Research & Development Institute for Food Bioresources-IBA Bucharest, 6 Dinu Vintilă Street, District 2, 021102 Bucharest, Romania; mioaranegoita@yahoo.com (M.N.); andreeahornet29@yahoo.com (G.A.H.)

**Keywords:** acrylamide, mitigation, French fries, home-made, citric acid, blanching, potatoes

## Abstract

The aim of this study was to investigate the influence of some pre-treatment applications toward acrylamide mitigation in potatoes fried in domestic conditions modeled after those found in Romania, by using a pan and a fryer. Before being fried in a pan, potato strips were treated in one of the following ways: soaked in cold water for 15, 60, and 120 min (a); soaked in hot water at different combinations of temperatures and durations (60, 70, 80 °C for 5, 10, 15 min) (b); soaked in a NaCl solution (c), and; in a citric acid solution (d) both solutions of 0.05% and 1% concentration for 30 min. For potatoes fried in a fryer, the (a) pre-treatment and soaking in water at 80 °C for 5, 10, and 15 min were applied. Untreated samples were used as a control. French fries were analyzed in terms of moisture and acrylamide content, color, and texture parameters. The pre-treatments applied reduced the acrylamide content in French fries by 4–97% when fried in the pan and by 25–47% when fried in the fryer. Acrylamide content of French fries was negatively correlated with L* parameter and moisture content and positively correlated with a* parameter. The pre-treatments applied can be used successfully by consumers to reduce acrylamide content.

## 1. Introduction

Potatoes (*Solanum tuberosum* L.) are widely cultivated around the world and are consumed in large quantities. In Europe, the harvested production of potatoes was 55.3 million tonnes in 2020. The largest producer of potatoes from Europe was Germany (21.2% of the EU total), followed by Poland (16.4%), France (15.7%), and the Netherlands (12.7%). In Romania, the share of EU harvested production in 2020 was 4.9%, generating 2.683 million tonnes [1]. Potato-based products are highly consumed foods in Romania, of which French fries and chips are on top of Romanians’ preferences.

During frying, potatoes suffer a lot of changes in their sensory attributes like surface color, texture parameters, and aroma. Additionally, during this thermal treatment, water evaporates, products absorb oil, starch gelatinization, and protein denaturation take place [2,3]. On the other hand, by frying potatoes acrylamide is formed, a chemical contaminant that is classified by the International Agency for Research on Cancer (IARC) as a “probable human carcinogen” [4]. Based on animal studies, the European Food Safety Authority (EFSA) confirmed previous assessments that acrylamide in foods may increase the risk of developing cancer for consumers of all ages [5]. It was concluded that dietary exposure to acrylamide is not a concern when it comes to non-neoplastic effects, but based on animal studies, when it comes to margins of exposure, acrylamide disturbs the neoplastic effects [5].

Acrylamide is the result of a Maillard reaction, the non-enzymatic browning reaction which takes place between reducing saccharides and amino acids, mainly asparagine, being present in all carbohydrate-rich food products. Acrylamide is produced at temperatures above 120 °C and in low moisture conditions. Acrylamide can also be formed through the acrolein and acrylic acid pathway. During the frying process of oils, glycerol degrades and is transformed into acrolein which interacts with asparagine and forms acrylic acid, the final result of frying being acrylamide formation [6].

Foods that contribute the most to the acrylamide intake in the diet differ from country to country, depending on eating habits and how food is processed and prepared [7,8,9,10,11]. French fried potatoes are frequently consumed both in households, in fast-food venues, and in restaurants and it is known that potato-based products are considered to be among the foods with the highest levels of acrylamide due to the high concentration of asparagine, which is naturally found in potatoes. The chemical composition of potatoes varies depending on the variety, growing area, soil conditions, agricultural practices applied, maturity at harvesting, and storage conditions [12]. The determinant factor in acrylamide formation is the reducing saccharides content, therefore, a potato variety with a lower reducing saccharides content leads to a lower acrylamide content in the final product [13]. In a previous study [14], it was shown that the acrylamide content of French fries is influenced by the home-cooking practices of frying potatoes, and consumers should be educated to use appropriate mitigation strategies to obtain safer products in terms of acrylamide content.

In order to ensure food safety, in the European Union, it is mandatory to reduce acrylamide in food products by establishing appropriate mitigation measures. In this regard, the representatives of the EU Member States of the European Commission (EC) adopted in 2017 Regulation 2158 laying down mitigation measures and benchmark levels for reducing the acrylamide presence in foodstuffs [15]. French fries can easily exceed the benchmark level set by the EU Regulation (500 μg/kg), and consumers and food business operators should apply mitigation strategies and adjust their processes in order to decrease the acrylamide content below the limit set by the Regulation.

This Regulation provides measures to reduce the level of acrylamide in certain foods, in order to achieve the lowest possible concentrations of acrylamide that are below the benchmark levels set by this legislation. The benchmark levels indicated in Regulation No. 2017/2158 are not regulatory limits or safety thresholds but are performance indicators used to verify the effectiveness of mitigation measures and ultimately food safety [15]. 

Food Drink Europe [16] proposed a toolbox with recommendations for individual manufacturers and food business operators on how to correctly implement the EU 2017/2158 to mitigate the acrylamide content. In 2016, the Food and Drug Administration (FDA) established a guide that presented recommendations to help growers, manufacturers, and also food business operators to mitigate the acrylamide content in food products [17].

Several strategies can be taken into consideration to reduce the acrylamide level of food products. The easiest way to mitigate the acrylamide content of potato-based products is to use raw materials with a low content of asparagine or to reduce saccharides, establish optimal storage conditions for potatoes, and apply pre-treatments before frying, modify the manufacturing recipes, and optimize the preparation conditions. Lately, enzymes and different inhibitors of acrylamide formation were studied such as asparaginase [18,19,20], antioxidant compounds, in particular, phenolic compounds [21], β-cyclodextrin and β-CD-carvacrol complexes from thyme oils [22], thiol compounds, and also different treatments like high-pressure processing [20], pulsed electric field [23], and gamma irradiation [24] which were applied to mitigate acrylamide content in potato-based products. However, by using these pre-treatments, the cost of these products will be higher. Several inexpensive and easy-to-use alternatives to mitigate acrylamide content are soaking potatoes in cold or hot water before frying [13,23,24,25] and soaking potatoes in different additives to lower the pH [26,27,28]. By blanching potatoes in water, the starch gelatinization is promoted and oil uptake during frying is reduced [23]. Additionally, by blanching, potatoes’ pectins are hydrothermally degraded and the middle lamellae and primary cell walls of potatoes are ruptured which results in the softening of potatoes [23]. By soaking potatoes in water alone or with different additives (citric acid, acetic acid, ascorbic acid, glycine, calcium lactate, calcium chloride, sodium acid pyrophosphate, NaCl, nicotinic acid), acrylamide precursors (asparagine and reducing saccharides) in potato strips are decreased through the transfer to the blanching water, and, as a consequence, acrylamide content is decreased [13,27,28,29]. Blanching potatoes at temperatures between 55–75 °C can improve the color and textural quality of French fries [13]. The lightness of potato products increases after applying the soaking pre-treatment [23,30], hardness decreases [23], while the chewiness is increased [23].

This study realized for the first time in Romania was conducted with the aim to investigate the influence of blanching and soaking potatoes in water and NaCl and citric acid solutions on the acrylamide content of French fries obtained from a variety of potatoes produced locally by simulating the conditions of consumers’ households (frying in a pan and a fryer). The pre-treatments selected to be applied in this study for evaluation of their effect on the acrylamide content were chosen to be: easy, inexpensive, commonly used by consumers, and highly efficient. 

Moreover, the study aimed to establish the correlations between the acrylamide content and moisture and color parameters of French fries, respectively. The results can provide very useful information for consumers and French fries manufacturers on what pre-treatments to use to reduce the acrylamide content in potatoes fried in a pan or a fryer.

## 2. Materials and Methods

### 2.1. Raw Materials and Auxiliary Materials

Potato tubers (*Solanum Tuberosum*, Queen Anne variety) harvested in 2019, with the label “special for frying” were purchased from a local supermarket in Bucharest, Romania. Potatoes were stored until analyses in a cold storage room, in boxes, at a temperature of 10 °C and air relative humidity was 80–90%.

Vegetable fats were used for frying potatoes: sunflower oil and refined, non-hydrogenated palm oil.

To obtain solutions for pre-treatment application on potato strips before frying, salt (NaCl) and citric acid were used.

### 2.2. Pre-Treatment Application

Potatoes brought to room temperature were washed, peeled, and then cut into strips. The slicing was done with a manual potato slicer (Zhejiang Yingxiao Industry and Trade Co, Jinhua, China), equipped with stainless steel knives and a slicing thickness of 9 × 9 mm chosen in the case of potatoes being fried in a pan. In order to choose the potato slicing for the fryer, several variants were tested: 7 × 7 mm, 9 × 9 mm, and 11 × 11 mm. Based on previous results, it was chosen to apply the pre-treatments for potatoes cut at 7 × 7 mm as these potatoes had the highest acrylamide content.

Immediately after cutting, potato strips were rinsed in the same amount of cold water for 30 s in order to remove the starch adhering to their surface. The potato strips were left to drain for 5 min in a stainless-steel sieve and then pre-treated with various solutions.

For potatoes fried in a pan, in order to simulate the domestic conditions, the potato strips were subjected to pre-treatments as follows: (a) soaking in cold tap water for 15, 60, and 120 min; (b) soaking in hot water preheated at temperatures of 60 °C, 70 °C, and 80 °C, with holding times of 5, 10, and 15 min; (c) soaking in NaCl solution of 0.05% and 1% concentration for 30 min; (d) soaking in a citric acid solution of 0.05% and 1% concentration with a holding time of 30 min.

For potatoes fried in a fryer, the potato strips were subjected to the following pre-treatments: (a) soaking in cold water for 15, 60, and 120 min; (b) soaking in hot water preheated at a temperature of 80 °C, with holding times of 5, 10, and 15 min.

For each pre-treatment variant, control tests were performed (noted as 0), without any treatment application prior to frying. All experiments were performed in duplicate.

After applying each pre-treatment, the samples were rinsed in a stream of cold water for 30 s, and then the potato slices were left to drain for 10 min in a stainless-steel sieve before frying.

### 2.3. Frying Conditions

#### 2.3.1. Frying in a Pan

To investigate the influence of pre-treatments applied to potato strips fried in domestic conditions, a ceramic pan was used, non-stick, 24 × 24 cm, height 15 cm, thickness 2.5 mm, provided with a sieve for frying potatoes. Sunflower oil was used to fry the potatoes, using an oil/potato ratio (*w*/*w*) of 1:1 (375 g oil and 375 g potatoes). The frying temperature was monitored using a digital food thermometer (BBQ thermometer, TP500) (measuring range: −50 °C to +350 °C; accuracy ± 1 °C), and when the oil temperature reached 120 °C, potatoes were immersed in oil. The frying end-point was chosen based on the color of control samples of French fries. In the case of potatoes pretreated with water, the golden-yellow color of the control sample was obtained after 5 min of frying, while for potatoes soaked in NaCl and citric acid solution this color was obtained after 7.5 min. Based on the frying time of the control sample it was set the frying time for the pretreated samples. The temperature was monitored at regular time intervals (1 min) and for all pre-treatments applied, it ranged between 120 and 130 °C. After frying, to remove the excess oil, the frying sieve was shaken and then the potatoes were left to dry on absorbent paper.

#### 2.3.2. Frying in a Fryer

For the strips fried in a fryer, an electric fryer (Hendi Blue Line, Rhenen, The Netherlands) made of stainless steel, with a capacity of 4 L and equipped with 2 detachable frying pans with an element warning light with a heating and safety thermostat to prevent overheating was used. Palm oil was used to fry the potatoes, using a 4:1 oil/potato ratio (*w*/*w*) (2536 g oil and 634 g potatoes). The frying temperature was adjusted to 190 °C and was monitored during frying, using the same digital thermometer. The frying time of the potato strips was 11 min.

Potato strips were placed on absorbent paper after frying. The experiments were performed in duplicate, using fresh oil, without reusing the oil in any experiment.

After frying, French fries were ground in a Büchi mixer (B-400, BÜCHI Labortechnik AG, Flawil, Switzerland) and stored at −25 °C until further analysis.

### 2.4. Chemicals and Solvents

Standard of native acrylamide of min. 97.7% purity purchased from Dr. Ehrenstorfer (LGC Group, North Charleston, SC, USA), and internal standard of acrylamide with labeled carbon atoms (1,2,3-13C), purity ≥ 98% (+100 ppm hydroquinone) purchased from Cambridge Isotope Laboratories (Andover, MA, USA) were used.

All chemicals, solvents, and reagents used in the analysis of French fries were of chromatographic purity. Potassium bromide was purchased from Merck (Darmstadt, Germany), hydrobromic acid 48% (ACS reagent) was from Alfa Aesar (Thermo Fisher Scientific, Waltham, MA, USA), and bromine 99.6% for analysis was obtained from Acros Organics (Geel, Belgium). Sodium thiosulfate pentahydrate was acquired from Scarlab S.L. (Barcelona, Spain), while sodium sulfate anhydrous AR, granular, and all solvents (n-hexane, ethyl acetate, and methanol) picograde for residue analysis were from LGC Promochem GmbH (Wesel, Germany). Triethylamine > 99.5% was purchased from Sigma-Aldrich (St. Louis, MO, USA).

Two solid-phase extraction (SPE) columns were used to purify the aqueous acrylamide extract: Isolute Multimode (1000 mg, 6 mL) and Isolute ENV^+^ (500 mg, 6 mL), supplied from Biotage (Uppsala, Sweden).

### 2.5. Chemical Analysis

The moisture content (%) of French fries was determined gravimetrically in an oven heated at 105 °C to constant weight for 24 h according to the AOAC method [31] after samples were ground in a Büchi mixer. The tests were performed in duplicate.

The pH of the NaCl and citric acid solutions used in the pre-treatments of potato strips before frying was determined with a pH-meter type 827 pH Lab (Metrohm, Herisau, Switzerland).

### 2.6. Color Analysis

The Konica Minolta colorimeter (Universal Software V4.01 Miniscan XE Plus) was used to determine the values of the CIELAB’76 color parameters of the French fries samples. For each sample, measurements were performed at 10 different points of ground French fries, and then the mean was calculated. CIELAB’76 color parameters were calculated: L*—the lightness intensity of the object on a scale from 0 to 100, where 0 represents black and 100 white; a* (green to red) and b* (blue to yellow) are the two color components ranging from –127 to 127 (−127 is pure green for component a* and pure blue for component b*; +127 represents pure red for component a* and pure yellow for component b*).

### 2.7. Texture Analysis

The texture properties of the French fries samples were measured with the Instron Texture Analyzer, model 5944 (Illinois Tool Works Inc., Glenview, IL, USA) equipped with a 50 N load cell. Ten strips of French fries were perforated once at room temperature with a 3 mm compression piston and transverse head speed set at 10 mm/min. For each sample, 10 measurements were performed. The firmness (hardness), expressed in N, defined as the maximum compressive force was calculated and the average of ten replicates was reported (Bluehill software, version 3.13, Norwood, MA, USA). Additionally, fracturability was measured.

### 2.8. Acrylamide Determination by GC-MS/MS

#### 2.8.1. Preparation of Stock and Working Solutions

The stock solutions of acrylamide and internal standard were prepared in ultrapure water to a concentration of 100 mg/L. Working solutions of acrylamide of concentrations of 10, 1, and 0.1 mg/L and a working solution of an internal standard of a concentration of 1 mg/L were prepared by diluting the stock solution with ultrapure water. All stock and working solutions were kept in a refrigerator at 4 °C.

#### 2.8.2. Preparation of the Calibration Solutions

The working solutions of acrylamide (10, 1, 0.1 mg/L) were diluted with ultrapure water to obtain calibration solutions of 0.05; 0.10; 0.20; 0.30; 0.40; 0.50; 0.75; 1; 2; 3 mg/L, all containing 1 mg/L of acrylamide 1,2,3-13C. These solutions were derivatized with KBr, HBr (pH 1–3) and saturated bromine-water solution (around 1.6%) on a shaking water bath set at a temperature below 4 °C, for at least 1 h. After the end of the derivatization reaction, bromine in excess was removed by adding around 100 mL of 1 mol/L sodium thiosulfate, until the yellow color disappeared. Extraction of dibromo derivative of acrylamide (2,3-dibromopropanal- 2,3-DBPA) was achieved with a 70 mL mixture of ethyl acetate and hexane (4:1, *v*/*v*).

The extract concentration was realized in two steps: by using a vacuum evaporation system (Rotovapor R-210, BÜCHI Labortechnik, Flawil, Switzerland) until 2 mL, followed by a drying process under a stream of nitrogen. The final residue was redissolved in 1000 μL ethyl acetate and 100 μL triethylamine. The final extract (2-BPA) was filtered through a 0.2 μm regenerated cellulose microfilter (17 mm diameter, Spartan 13RC, Whatman GmbH, Dassel, Germany) directly in a vial and subjected to acrylamide analyses by GC-MS/MS.

For acrylamide quantification, two calibrations curves in the ranges 0.05–0.5 mg/L and 0.4–3 mg/L, respectively, were used.

#### 2.8.3. Sample Preparation for Acrylamide Analysis

For the acrylamide analysis, 1 g of homogenized French fries was weighted in 50 mL centrifuge tubes. The sample was spiked with 440 μL internal standard solution (1 mg/L) and 20 mL water and 10 mL n-hexane was added to the centrifuge tubes. Samples were vortexed for 60 min at ambient temperature and then were centrifugated at 5 °C, 6000× *g* for 20 min (5804R Eppendorf centrifuge with cooling, Eppendorf, Hamburg, Germany). The aqueous extract was collected (10 mL) and for the clean-up, the SPE was performed by using the HyperSep Universal Vacuum Solid Phase Extraction Manifold (Thermo Fisher Scientific, Waltham, MA, USA) and two types of SPE columns were used. The first SPE column (Isolute Multimode) was conditioned with MeOH (3 mL) and water (3 × 4 mL). The aqueous extracts (10 mL) were loaded onto the column and the eluate was collected. The second SPE column (Isolute ENV^+^) was conditioned with MeOH (5 mL) and water (5 mL). The extract from the first column was loaded and after it was rinsed with water (4 mL) and then the eluate and the rinsing solvent were discarded. After this step, the second column was loaded with 5 mL of 60% MeOH in water in which acrylamide was collected.

After purification, the acrylamide extract was derivatized in the same manner as the derivatization steps described for the preparation of the calibration solutions and the final residue was redissolved in 400 µL ethyl acetate and 40 µL triethylamine.

#### 2.8.4. GC-MS/MS Analysis

The calibration solution and the derivatized extract samples were analyzed using a gas-chromatograph (TRACE GC ULTRA) coupled with a triple quadrupole mass spectrometer (TSQ Quantum XLS) from Thermo Fisher Scientific (San Jose, CA, USA). The chromatographic separation was achieved on a capillary column, TraceGOLDTM TG-WaxMS (30 m × 0.25 mm i.d. × 0.25 μm film thickness) acquired from Thermo Fisher Scientific (Waltham, MA, USA). For sample injection, an automatic injector (Right PTV) and a TriPlus AS autosampler (Thermo Fisher Scientific, San Jose, CA, USA) were used. A volume of 1 μL sample was injected onto the column, and the following chromatographic conditions were used: oven temperature was set to 65 °C and held for 1 min, on the first ramp it increased with 15 °C/min to 170 °C, on the second ramp with5 °C/min to 200 °C, and on the third ramp with 40 °C/min to 240 °C and held for 15 min at this temperature (total run time: 30 min). The transfer line of the GC-MS was held at 245 °C and the source temperature at 230 °C. Helium (1.6 mL/min, constant flow) was used as carrier gas and argon was used as collision gas (min. 99.9995% purity).

The mass selective detector was set in the electron impact ionization operation mode (EI^+^) and Selected Reaction Monitoring mode (SRM), selecting characteristic fragments of each derivatized acrylamide, 2-BPA, and derivatized internal standard, 2-BP(^13^C3)A. The fragmentation of the precursor ions with *m*/*z* 151 and 154 was achieved with argon (1 mTorr), leading to the formation of product ions (daughter) with *m*/*z* 70 (2-BPA) and 73 (2-BP(^13^C3)A), being used for quantification.

#### 2.8.5. Validation Parameters

For the method validation, several parameters were assessed: linearity, linearity range, sensitivity (limit of detection—LOD, and limit of quantification—LOQ), selectivity, precision, accuracy, recovery, and measurement uncertainty as described by Negoiță et al. [32]. 

Calibration curves and linearity were verified by the method of least squares in the range 0.05–0.5 mg/L and 0.4–3 mg/L, choosing the method of the internal standard. The calibration curves were obtained by plotting the peak area ratio of 2-BPA to the 2-BP(^13^C3)A derivative against the concentration of 2-BPA. The correlation coefficients (R^2^) were higher than 0.998.

The correlation coefficient (R^2^), tested in the linearity range 30.87–2636.06 µg/kg, was higher than 0.999.

LOD and LOQ were determined by serial dilution of a reference material (French fries—T3085QC, 229 µg/kg assigned value |z| ≤ 2: 137–320 µg/kg) and were set at 10.29 and 30.87 µg/kg, respectively. Selectivity was investigated by using the SRM detection and by using the internal standard method which led to a specific analysis.

The method was characterized by good accuracy, expressed as a relative standard deviation, under repeatability, reproducibility, and intermediary precision, as follows: 1.13–4.26%, 1.81–7.21%, and 1.19–8.84%, respectively.

The method’s precision and accuracy were demonstrated for potato-based products by the results obtained in two proficiency tests launched by the Food Analysis Performance Assessment Scheme (FAPAS), yielding a z-score of −0.8 for French fries precooked (test 3095/2019), and 0 for potato crisp test material (test 3099/2020). Recovery ranged between 85.64% and 109.22%. The method uncertainty established by the uncertainty budget was ± 17.5%.

#### 2.8.6. Acrylamide Reduction

The percentage of acrylamide reduction after applying the pre-treatments was calculated with the formula:

%AA reduction = (AA_c_ – AA_t_) × 100/AA_c_ (1)

where AA is acrylamide, AA_c_ is the acrylamide content of the control sample, and AA_t_ is the acrylamide content of the pretreated samples.

### 2.9. Statistical Analysis

Results were statistically analyzed by using Minitab statistical software version 20. One-way analysis of variance (one-way ANOVA) followed by Tukey’s test was used to evaluate the statistical significance between samples. The chosen level of significance was set at *p* < 0.05. Pearson correlation was performed to determine the relationship between the acrylamide content and moisture content, color parameters, and hardness, respectively. All results were expressed as mean ± standard deviation.

## 3. Results and Discussion

Frying potatoes is one of the oldest and most popular methods of cooking. The frying conditions of fast cooking lead to increases in the rate of heat transfer, causing textural changes and the development of flavors. High heat transfer rates are largely responsible for the development of the desired sensory properties in French fries but at the same time, contribute to acrylamide content.

In this study, two approaches to reducing acrylamide content in French fries were studied including soaking in water and soaking in NaCl/citric acid solutions.

### 3.1. Effect of Water Pre-Treatments Applied on the Acrylamide Content in French Fries

Asparagine and reducing saccharides are the main precursors in acrylamide formation and its reduction is one of the important factors in acrylamide mitigation [27,28]. One of the easiest pre-treatments that can be applied by consumers and food business operators in order to reduce acrylamide content in French fries without substantial costs is to soak and maintain potato strips in cold water or to simulate the conditions of the product industry and blanch potatoes in hot water before frying. In doing this, the precursors will transfer to the wash water. Due to this consideration, one of the aims of this study was to investigate the effect of soaking potato strips in water on the acrylamide content of French fries.

#### 3.1.1. Potatoes Fried in a Pan

Acrylamide content of French fries soaked in cold and hot water was determined in order to evaluate the efficacy of the pre-treatment applied to potato strips before frying to mitigate acrylamide content. To simulate the domestic conditions, potato strips were fried in sunflower oil at a temperature under the temperature of 175 °C recommended by the EC [15]. 

**Figure 1:**
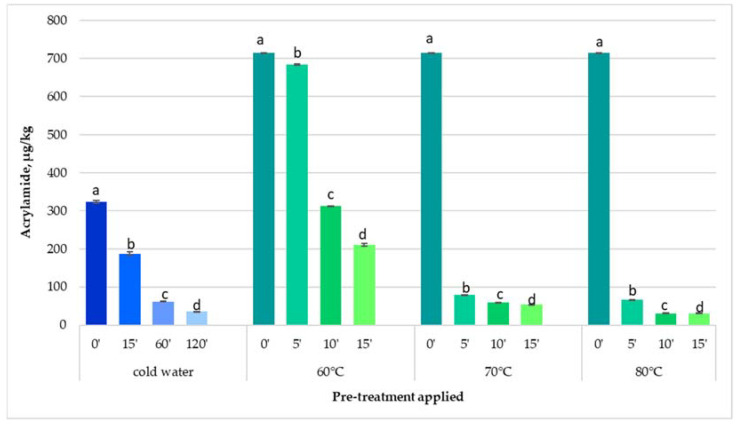
Effect of cold and hot water applied on potato strips on the acrylamide content of French fries fried in a pan. Values followed by different letters in the same column for each pre-treatment are significantly different (*p* < 0.05).

Soaking potato strips in cold water before frying and keeping them for different times led to reductions in acrylamide content compared to the control sample, without the application of pre-treatments. Figure 1 shows the results of acrylamide content of French fries fried in a pan after applying cold and hot water pre-treatments. All tested pre-treatments gave a positive effect on the reduction of acrylamide content.

Potato strips soaked in water at room temperature for 15, 60, and 120 min, respectively, and fried in a pan, determined significant reductions of acrylamide content by about 42% (187.40 µg/kg), 81% (61.71 µg/kg), and 89% (35.77 µg/kg), respectively, compared to the control samples (323.41 µg/kg). By increasing the soaking time in cold water, the acrylamide content of French fries significantly decreased (*p* < 0.05).

Blanching was another pre-treatment applied to potato strips to reduce acrylamide content in French fries, a process that can change the microstructure of potato strips, being responsible also for enzyme inactivation. Blanching of French fries is usually carried out in hot water at a temperature between 60–85 °C for 10–30 min [33].

Experiments with potatoes blanched in water at 60–80 °C were performed two weeks later after the experiments with cold water. Potatoes were stored at 10 °C in dark places as recommended by the EC [15]. As can be seen in Figure 1, the acrylamide content of the untreated sample increased significantly after storage compared to the untreated sample analyzed in the experiments with cold water. This result could be due to the acrylamide content being influenced by the storage conditions and time, during this step the reducing saccharides content of tubers increased because of the senescent sweetening, and also the potato starch degraded [34]. 

By soaking the potato strips in hot water at different temperatures and time periods, the acrylamide content decreased significantly (*p* < 0.05) compared to the control sample (Figure 1). Additionally, when comparing the same time of soaking potatoes at different temperatures of water, significant differences (*p* < 0.05) were obtained between the acrylamide content of French fries.

The decreases in acrylamide content were proportional to the hot water pre-treatment conditions: 4%, 56%, 71% at 60 °C/5, 10, 15 min; 89%, 92%, 93% at 70 °C/5, 10, 15 min; and 91%, 96%, 97% at 80 °C/5, 10, 15 min. Blanching potato strips in water at 80 °C for 10 and 15 min, respectively, had the greatest effect on reducing the acrylamide content in French fries. Thus, blanching at temperatures of 70 °C and 80 °C and maintaining the potato strips for 10–15 min, had a higher efficiency of reducing the acrylamide content (92–97%), compared to the temperature of 60 °C (56–71%), the same duration of pre-treatment.

Blanching the potato strips in water at 70 °C for 10 min caused the same acrylamide reduction (about 89%) as maintaining the potato strips in cold water for 120 min. Thus, blanching potatoes at temperatures of 70 °C and 80 °C for a short period of time, 10–15 min, had a higher efficiency in reducing acrylamide content (89–96%) compared to maintaining potato strips in cold water for longer periods of time.

Similarly, Liyanage et al. [28] showed that by blanching three varieties of potatoes and then frying them at 180 °C for 5 min, the acrylamide precursors decreased, and similarly, the acrylamide content of French fries decreased. The highest acrylamide decrease was obtained for potato strips blanched in distilled water (19–59%), compared with the blanching in solutions with different additives. In a study realized by Zhang et al. [23], potatoes were blanched in water at 100 °C for 4 min and a reduction of acrylamide content in French fries of 18% was obtained, lower than the one obtained in our study.

#### 3.1.2. Potatoes Fried in a Fryer

**Figure 2:**
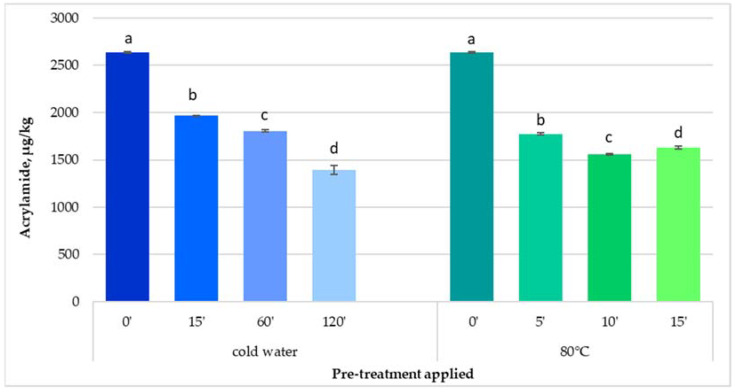
Effect of cold and hot water applied on potato strips on the acrylamide content of French fries fried in a fryer. Values followed by different letters in the same column for each pre-treatment are significantly different (*p* < 0.05).

When frying potato strips in a fryer after potatoes were soaked in cold water for 15, 60, and 120 min, respectively, the acrylamide content of French fries was reduced by about 25% (1973.69 µg/kg), 31% (1809.19 µg/kg), and by 47% (1394.26 µg/kg), respectively, compared to the control sample (2636.06 µg/kg) (Figure 2).

Similar results were obtained by Burch et al. [35] who showed that when soaking potato slices in cold water for 30 and 120 min, respectively, the acrylamide content in French fries decreased by 35–50%.

The acrylamide content of French fries fried in a fryer was significantly higher (*p* < 0.05) compared to the ones fried in a pan. This increase in acrylamide content was the result of the higher temperature used, the potato surface to oil ratio, and also the size of potato strips influenced the acrylamide content. It is known that a smaller slice size of potatoes determines a higher acrylamide content [16,36]. During this pre-treatment, acrylamide precursors were transported from the surface layers to the pre-treatment medium of the potato strips, thus reducing the acrylamide content [37].

Even though the acrylamide content was diminished by soaking potatoes in cold water before frying, the content exceeded the benchmark level set by EC [15].

Based on the fact that for potatoes cooked in a pan the highest reduction of acrylamide content was obtained for potatoes soaked in water at 80 °C, this temperature was chosen as pre-treatment of potatoes fried in a fryer. By keeping the potato strips in water at 80 °C for 10 and 15 min, respectively, before frying, the acrylamide content was reduced by about 41% (1561.66.61 μg/kg) and 38% (1630.82 μg/kg), respectively, compared to the control sample (2636.06 μg/kg). The blanching time did not have a high influence on the acrylamide content of French fries, with 10 min of blanching being enough to mitigate acrylamide content by 41%. The acrylamide level formed in the control sample was about 5-fold higher compared to the benchmark level of 500 μg/kg set by EC [15].

Similarly, Abboudi et al. [24] showed that by applying a blanching treatment to potato slices, at a temperature of 85 °C for 5 min, before frying in the fryer (170 °C, 5 min), a decrease of 61% in acrylamide content of fried potatoes compared to the control sample was obtained. When Zuo et al. [18] studied the blanching treatment with water at 80 °C for 0, 1, 2, 5, and 10 min on the level of acrylamide formed in French fries, it was observed that the acrylamide content decreased to 1323 µg/kg for 1 min and to 967 µg/kg for 10 min of blanching, respectively, compared to the control sample (1592 µg/kg). The authors concluded that the slight reduction in acrylamide content may be caused by the partial elution of acrylamide precursors during blanching. The effect of this pre-treatment on acrylamide reduction depends mainly on the temperature and duration of blanching. In a study realized by Zhang et al. [30], it was shown that by blanching potatoes at 65–85 °C for 2 to 10 min, the acrylamide content of potato chips was influenced more by the blanching time than blanching temperature, the reducing saccharides, asparagine and acrylamide content of potatoes was reduced gradually with the increasing of blanching time.

Additionally, in the study realized by Shojaee-Aliabadi et al. [13], it was shown that by blanching 3 varieties of potatoes (Agria, Sante, and Savalan) at 75 °C for 9 min, and 83 °C for 2.5 min, respectively, there was a 64–77% decrease of reducing saccharides, and a 40–66% reduction of asparagine content was obtained compared to the control sample. These reductions were correlated with the decrease in acrylamide content formed in the chip samples. 

Several authors have tried to optimize the blanching conditions in order to maximize the extraction of reducing saccharides and asparagine while maintaining the final characteristics of the product [12,37]. They concluded that the application of a temperature of about 70 °C and a blanching time of about 15 min can be considered an effective technique for reducing acrylamide content in French fries, because these pre-treatments achieve not only the reduction of acrylamide content but also the improvement of certain quality attributes, such as the firmness of the potatoes and the decrease of the consumption of oil used for frying. In addition to time and temperature, the concentration of soluble components extracted from potato strips during this continuous process, will influence the efficiency of saccharides extractability and, therefore, will influence the acrylamide content in potato products. Extreme blanching conditions lead to texture modification and loss of nutrients. However, the continuous replacement of blanching water with freshwater is not feasible from an economical point of view [38].

### 3.2. Effect of Soaking in NaCl/Citric Acid Solutions Pre-Treatments Applied on the Acrylamide Content in French Fries

It is known that another way to reduce acrylamide content is by decreasing the pH of potatoes, thus in this study, NaCl and citric acid which are widely known as food additives were used for this purpose. In order to evaluate the effect of these preservative agents on the acrylamide content, potato strips were soaked in NaCl and citric acid solutions of 0.05% and 1% concentration for 30 min. By using this pre-treatment, the pH was reduced which determined asparagine protonation, with this further reaction, reducing saccharides were being blocked, decreasing the acrylamide formation. Compared to soaking in water where the reducing saccharides and asparagine content decrease, in this case, the mechanism to decrease acrylamide content was different, with these preservatives changing the heat transfer and reducing the oil uptake [26]. In the case, of NaCl, another explanation is that this additive has a catalytic effect on the polymerization reaction which takes place during heating of reducing saccharides and asparagine and can accelerate acrylamide mitigation [39]. Through polymerization reaction, polyacrylamide is formed, a biologically nonactive high-molecular compound, resulting in the decrease of acrylamide content [40].

**Figure 3:**
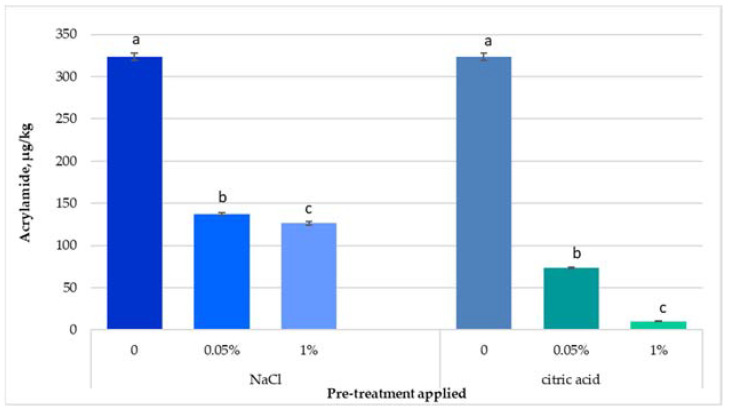
Effect of NaCl and citric acid solutions applied on potato strips on the acrylamide content of French fries fried in a pan. Values followed by different letters in the same column for each pre-treatment are significantly different (*p* < 0.05).

Soaking potato strips in NaCl solution reduced the pH from 7 for the control sample to 6.77 for 0.05% NaCl solution and to 6.49 for 1% NaCl solution, respectively, resulting in a decrease of the acrylamide content by up to 57% (137.55 μg/kg) and 61% (126.45 μg/kg) compared to the control sample (323.41 μg/kg) (Figure 3). The concentration of the NaCl solutions did not generate unwanted flavors, tastes, or color in the end product.

Water activity at the surface of potato strips is an important factor that limits the acrylamide content during frying. By increasing the NaCl concentration, the water activity decreases [37].

In a study realized by Kolek et al. [41], it was shown that NaCl can decrease acrylamide content up to 40% in an asparagine/glucose model system through polymerization reaction which is accelerated by this additive. Sansano et al. [27] showed that pre-treatment of potatoes with 2% NaCl, for 60 min at room temperature led to a 78% reduction in acrylamide content in French fries. Adding NaCl before the frying step could increase the rate of oil degradation on subsequent frying. This could lead to a higher rate of oil change in the industrial frying plant, causing additional production costs. Interestingly, NaCl has reduced the final acrylamide content, which was the result of the increase of moisture content, determining a reduced absorption of oil when frying potatoes. This fits perfectly with the current trend of consumers wanting to consume healthy, low-fat products to eliminate obesity [42].

Several authors studied the effect of other salts on acrylamide content in French fries. Kalita and Jayanty [43] studied the effect of salt of vanadyl sulfate on acrylamide content in French fries, and after immersion of potato strips in 0.001, 0.01, and 0.1 M vanadyl sulfate for 60 min and then frying the potatoes at 180 °C for 2 min, a reduction of acrylamide content of 30.3%, 53.3%, and 89.3%, respectively, was obtained. Similar to our study, results showed that the reduction in acrylamide content was correlated with the decrease in the pH of the samples soaked in salt solutions and with the increase of the concentration of the salt solution.

Immersion of potato strips in citric acid solution inhibited the formation of acrylamide, by significantly decreasing the pH value in control samples from 7 to 3.99 for 0.05% citric acid solution, and to 2.35 for the 1% citric acid solution, respectively, determining a significant reduction in acrylamide content by about 77% (73.44 μg/kg) and 97% (<LOD), respectively, compared to untreated samples. Similarly, Sansano et al. [27] showed that by immersing potatoes in 1% and 2% citric acid solutions prior to frying, the acrylamide content was reduced by 77 and 91%, respectively. In a study realized by Huang et al. [29], acrylamide content in French fries decreased by 56.8% when dipping potato strips in a 1% aqueous solution of citric acid. Acrylamide inhibition might be the effect of glyoxal and methylglyoxal decrease, α-dicarbonyl compounds which are precursors of acrylamide formation [29].

In a study realized by Jung et al. [44], it was shown that by immersing potato slices in 1% and 2% citric acid solutions for 1 h before frying, the pH was reduced from 6.2 to 5.2 and 4.9, respectively, and determined a 73.1% and 79.7% decrease of acrylamide content in French fries. Organic acids are known for their attenuating effect in the formation of acrylamide at low pH, due to the protonation of amino groups of asparagine. This would block the nucleophilic addition of the amino group of asparagine with a carbonyl compound, preventing the formation of the Schiff base, an intermediary product in the Maillard reaction. By lowering the pH, the acrylamide formation will be inhibited and the protonation of the α-amino group of asparagine is blocked so that it can no longer be involved in carbonyl-source nucleophilic addition reactions [45].

The acrylamide formation is limited by the pH, a low pH of the reaction mixture determines a decrease of acrylamide content [43], thus a higher reduction in acrylamide level in potatoes pre-treated with citric acid solution compared to the ones soaked in NaCl solution can be attributed to a lower pH. In a study realized by Kita et al. [46], it was shown that pH plays an important role in acrylamide formation in processed potato products, with the acrylamide content being reduced by 90% and 50% in potato chips, respectively, by immersing the potatoes in solutions of acetic acid and citric acid, respectively.

### 3.3. Changes in Moisture Content

Acrylamide is formed at low moisture conditions, so the moisture content is an important parameter to evaluate the pre-treatment effect on the acrylamide content of French fries. The moisture content was influenced by the pre-treatment applied. The moisture content of French fries pre-treated samples was higher than the moisture content of control samples, without any pre-treatment. Results of moisture content are presented in Table 1 and Table 2.

The moisture content of French fries that were soaked in cold water for 60 and 120 min was significantly different (*p* < 0.05) than the moisture content of untreated French fries or the ones soaked in water for 15 min.

When potato strips were blanched in water at 60–80 °C and maintained in it for 5–15 min before frying in a pan, the moisture content increased for all samples analyzed. By blanching potatoes at temperatures between 55–70 °C, the porosity of potato strips is reduced by the activation of pectinesteraze and, as a consequence, the oil uptake decreases [47].

When potato strips were fried in a fryer, the moisture content was significantly lower (*p* < 0.05) than the moisture content of French fries fried in a pan. The highest moisture loss was determined for potatoes deep-fried, this evaporation is attributed to the higher temperature used for this processing conditions, the moisture decrease taking place primarily in the outer layer of products [48]. During frying, water from the potato structure is replaced by oil absorption, these parameters being negatively correlated [49]. 

The moisture content of French fries deep-fried determined an acrylamide content higher than the benchmark level set by regulation in force, knowing that a moisture content below 40% determines a higher content of acrylamide [50].

When NaCl and citric acid solutions were used, the moisture content increased with the increase of solution concentrations.

### 3.4. Changes in Color Parameters

During frying, the Maillard reaction occurs and induces changes in the crust and implicitly in the color of French fries, with brown-colored melanoidins being formed [51]. The Maillard reaction depends on the acrylamide precursors concentration in potatoes which in this study was influenced by the pre-treatment applied. The color parameter is a reliable indicator of the French fries’ safety when it comes to acrylamide content, the acrylamide analysis could be replaced by color measurement [15].

Pre-treatment with cold water applied determined the leaching of reducing saccharides and asparagine in the water in which potatoes were soaked. Blanching, as well, determines the leaching of acrylamide’s precursors involved in the non-enzymatic browning reaction, which are associated with electrolyte leakage of potato strips and, therefore, the color development of French fries was reduced [23]. The color parameters of French fries after different pre-treatments are shown in Table 3 and Table 4. After applying the pre-treatments, the color parameters of French fries were significantly different compared to the control sample. For all pre-treatments applied, the lightness of French fries increased compared to the control sample.

Potato strips soaked in water for a longer time (120 min) and fried in a pan or a fryer led to the lowest acrylamide content and the highest values of L*.

In the case of blanched potatoes before frying, as the blanching temperature increased, lighter colors of French fries were obtained. After blanching potatoes, with the exception of potatoes blanched at 60 °C for 5 and 10 min, the value of a* parameter was smaller, with the products being less red. Similarly, Zhang et al. [30] showed that the blanching process decreases the a* values of potatoes.

Keeping the potato strips in a NaCl solution of 0.05% and 1% concentration resulted in a lighter color of French fries prepared in a pan, compared to those that were kept in cold or hot water, for the same duration of time.

Soaking potatoes in salt or citric acid solutions brightened the French fries samples, increasing the value of the L* parameter, the highest value was obtained for the samples on which these pre-treatments were applied. Regarding the a* parameter, in the case of potatoes soaked in NaCl solution, French fries tend to become less red, while in the case of the citric acid solution, the value of a* parameter decreased with increasing the solution concentration. 

### 3.5. Changes in Texture Parameters

Texture is one of the most important attributes of sensorial properties of French fries. The influence of pre-treatments with cold and hot water applied to potatoes fried in a pan on the texture attributes of French fries was analyzed and results are presented in Table 5. 

The hardness or firmness of French fries was expressed as the maximum penetration force of the piston into the potato strips (N) and this force gradually increased as the duration of soaking in cold water increased, French fries being crispier. French fries that were soaked in cold water for 120 min presented the highest hardness (0.58 N), therefore, this pre-treatment led to crispier French fries. The untreated sample showed the lowest hardness (0.34 N), with the potatoes having a softer texture.

For all potato strips kept in water before frying, the compression at maximum force of the French fries samples decreased with the increase of the time to keep the potato strips in water, the lowest value was obtained for the sample kept in cold water for 120 min (1.57 mm).

For the hardness attribute, French fries that were not subjected to blanching pre-treatment showed the highest value of hardness, potatoes being crispier. Blanching at a high temperature can determine a higher oil absorption during frying, a change of texture parameters, and a loss of firmness compared to untreated samples [13].

As the soaking time of potato strips in hot water increased, this parameter, measured in the maximum penetration force of the potato strip decreased, the French fries having a lower hardness, being softer due to water absorption, which is also correlated with an increase in the moisture content of the samples. The softening of potato tissues and implicitly the softening of French fries can be caused by the degradation of pectin and also by gelatinization and dehydration of starch [23].

As the blanching time of potato strips increased, the compression at maximum force decreased, and the potato strips became softer, with lower resistance to piston penetration.

Another texture parameter determined was the fracturability, which is known to be the force recorded at the breakage of the sample [52]. By soaking potatoes in cold or hot water prior to frying, no statistical difference in the fracturability of French fries samples compared with the untreated samples was recorded, this parameter slightly increasing for both pre-treatment with water.

### 3.6. Correlation between Acrylamide Content and Moisture Content

During the frying process, the moisture evaporates on the surface of the potatoes and oil uptake occurs. 

The moisture content of French fries was influenced by the pre-treatment applied to potato strips, during this process, the porosity of potato strips increases which influence the evaporation process. Pearson correlation was performed in order to establish the relationship between acrylamide and moisture content and the results are presented in Table 6. In order to evaluate the strength of correlation the following interpretation for correlation coefficient (r) was used: little if any correlation (r < 0.3), low correlation (0.3 < r < 0.5), moderate correlation (0.5 < r < 0.7), high correlation (0.7 < r < 0.9), and very high correlation (0.9 < r < 1) [53].

The moisture content is directly correlated with the acrylamide content of products, which is known to be formed at a temperature above 120 °C and low moisture conditions [5]. High and very high correlations were registered between the acrylamide content of French fries pre-treated with both cold and hot water and the moisture content. By blanching potatoes, starch from the surface of potato strips gelatinize and, therefore, oil absorption is reduced, with French fries having a higher moisture content.

Negative, very high, and significant correlations were obtained between moisture content and acrylamide content of French fries pre-treated with cold water and fried in the fryer (r = −0.993, *p* < 0.01) and the ones blanched at 60 °C and 80 °C and fried in a pan (r = −0.955; −0.994, *p* < 0.05).

In the case of French fries pre-treated with NaCl and citric acid solutions, moderate (r = −0.460) and high correlations (r = −0.706) were obtained between the acrylamide content and moisture content.

### 3.7. Correlation between Acrylamide Content and Color Parameters

The correlation between acrylamide content and L* and a* parameters of French fries were determined, and the results are presented in Table 6. A lower acrylamide content of French fries is directly related to an increase in brightness and a decrease of redness [51,54].

A negative linear correlation between acrylamide and the L* parameter (lightness) was determined for all pre-treatments applied (r = −0.886 ÷ −0.998).

The brightness of French fries pre-treated with cold water increased significantly (*p* < 0.05) as the duration of soaking the potato strips increased being correlated with the decrease of the acrylamide content. By applying the pre-treatment with cold water, by using a pan and a fryer, a significant negative correlation was found between the acrylamide content of pre-treated potatoes and L* parameters, while significant positive correlations with a* parameter, respectively, were found.

These results were also confirmed by studies conducted by Cruz et al. [2] who showed that the color parameter L* depends on the duration of keeping the potato slices in cold water and that by applying this treatment, the content of reducing saccharides decreased, saccharides that contribute to the Maillard reaction and implicitly to the formation of color products, causing the increase of the products’ brightness. Additionally, in a study realized by Burch et al. [35], it was shown that as the duration of keeping the potato slices in cold water increased, they turned white, causing an increase in the value of the L* parameter, which was correlated with the decreased in acrylamide content (R^2^ = 0.70).

When applying hot water pre-treatment there is a good linear correlation of acrylamide content with color parameters, represented by brightness (L*) and redness (a*). As the temperature and duration of blanching potatoes increased, the acrylamide content was lower, and the potato strips became lighter in color, resulting in an increased value of the L* parameter and a decrease of the value of a* parameter. In a study realized by Zhang et al. [30], it was found that a linear correlation between the acrylamide content of potato chips and a* parameter (R^2^ = 0.839), but the correlation with L* value was low (R^2^ = 0.375).

In the case of using the pre-treatment with NaCl solution, negative correlations were observed between color parameters, L* and a* and acrylamide. The obtained results are also confirmed by Santis et al. [55] who showed that by keeping the potato slices in a salt solution, the color was improved, and the potatoes became lighter in color, compared with the control samples.

When citric acid solutions were applied, a good linear correlation was obtained with r = −0.888 for L* and 0.986 for a* parameter, respectively.

### 3.8. Correlation between Acrylamide Content and Hardness Parameter

When analyzing the relationship between the acrylamide content of French fries pre-treated with cold and hot water, fried in a pan, and the hardness, a very high negative correlation was obtained for samples treated with cold water (Table 7). When blanching was applied, positive correlations were obtained between the acrylamide content and hardness of French fries.

## 4. Conclusions

By applying some pre-treatments of potatoes before frying, like the soaking of potato strips in cold/hot water, in NaCl and citric acid solutions, a reduction of the acrylamide content can be achieved between 4–97% compared to control samples (not pre-treated).

The applied strategies to mitigate acrylamide content in French fries samples have been found to be efficient.

The efficiency of the four pre-treatments applied to the potato strips, before frying in a pan, in order to minimize the acrylamide content in French fries were as follows: cold water- 42–89%, hot water- 4–97%, NaCl solution- 57–61%, and citric acid solution- 77–97%. For French fries prepared in a fryer, a reduction of acrylamide content of 25–47% was obtained when cold water was applied, and 57–61% when water at 80 °C was used.

A negative correlation was found between acrylamide content and moisture content of French fries. Results showed that there is a strong correlation between the acrylamide content of French fries and color parameters L* and a*. A lower acrylamide content means a lighter color of French fries and a lower value of the redness parameter. By soaking potatoes in cold water, the hardness of French fries increased, while by soaking in hot water, products became softer.

The application of the pre-treatments for the reduction of acrylamide levels in French fries and food products, in general, must be done accordingly, so that the nutritional quality, safety, and sensory attributes are not affected in order to be accepted by consumers.

## Figures and Tables

**Table 1:** Moisture content of French fries pre-treated with water.

Pre-Treatment	Moisture Content (Mean ± sd), %/Pre-Treatment Time			
Time (min)	0	15	60	120
**CW-pan**	62.86 ± 0.59 ^b^	62.23 ± 0.86 ^b^	67.71 ± 0.48 ^a^	67.81 ± 0.37 ^a^
**CW-fryer**	9.61 ± 0.07 ^d^	15.74 ± 0.54 ^c^	19.07 ± 0.41 ^b^	22.96 ± 0.72 ^a^
**Time (min)**	**0**	**5**	**10**	**15**
**60 °C-pan**	58.64 ± 2.33 ^a,b^	57.8 ± 2.04 ^b^	61.63 ± 0.13 ^a,b^	64.81 ± 1.22 ^a^
**70 °C-pan**	58.64 ± 2.33 ^b^	65.55 ± 0.11 ^a^	63.27 ± 1.43 ^a,b^	66.38 ± 1.19 ^a^
**80 °C-pan**	58.64 ± 2.33 ^b^	64.18 ± 0.67 ^a^	66.61 ± 0.34 ^a^	68.77 ± 0.25 ^a^
**80 °C-fryer**	9.61 ± 0.07 ^b^	14.17 ± 0.74 ^a^	15.84 ± 0.07 ^a^	15.80 ± 0.33 ^a^

CW—cold water, sd—standard deviation. Values followed by different letters in the same line for each pre-treatment are significantly different (*p* < 0.05).

**Table 2:** Moisture content of French fries pre-treated with NaCl and citric acid solutions.

Pre-Treatment	Moisture Content (Mean ± sd), %/ Solutions Concentration		
Solution	0	0.05	1
**NaCl**	62.86 ± 0.59 ^b^	64.66 ± 0.13 ^a^	65.39 ± 0.28 ^a^
**Citric acid**	62.86 ± 0.59 ^b^	64.22 ± 0.30 ^b^	66.58 ± 0.57 ^a^

sd—standard deviation. Values followed by different letters in the same line for each pre-treatment are significantly different (*p* < 0.05).

**Table 3:** Color parameters (L*, a*, b*) of French fries pre-treated with water.

Pre-Treatment	Color Parameters/Pre-Treatment Time											
	L* (Mean ± sd)				a* (Mean ± sd)				b* (Mean ± sd)			
**Time (min)**	**0**	**15**	**60**	**120**	**0**	**15**	**60**	**120**	**0**	**15**	**60**	**120**
**CW-pan**	60.37 ± 0.03 ^d^	64.72 ± 0.03 ^c^	70.70 ± 0.01 ^b^	71.32 ± 0.05 ^a^	7.67 ± 0.01 ^a^	5.77 ± 0.01 ^b^	2.63 ± 0.01 ^c^	1.78 ± 0.03 ^d^	26.33 ± 0.03 ^d^	27.44 ± 0.04 ^a^	27.31 ± 0.03 ^c^	27.40 ± 0.01 ^b^
**CW-fryer**	61.06 ± 0.02 ^d^	63.45 ± 0.07 ^c^	64.08 ± 0.11 ^b^	65.95 ± 0.07 ^a^	11.06 ± 0.03 ^a^	8.06 ± 0.08 ^c^	8.42 ± 0.05 ^b^	6.95 ± 0.04 ^d^	42.21 ± 0.04 ^a^	36.71 ± 0.11 ^d^	38.89 ± 0.07 ^b^	37.53 ± 0.10 ^c^
**Time (min)**	**0**	**5**	**10**	**15**	**0**	**5**	**10**	**15**	**0**	**5**	**10**	**15**
**60 °C-pan**	57.45 ± 0.04 ^d^	58.30 ± 0.03 ^c^	59.92 ± 0.02 ^b^	64.52 ± 0.03 ^a^	7.55 ± 0.03 ^c^	8.88 ± 0.03 ^a^	8.01 ± 0.01 ^b^	6.33 ± 0.01 ^d^	27.30 ± 0.04 ^a^	25.49 ± 0.04 ^d^	25.58 ± 0.01 ^c^	26.66 ± 0.03 ^b^
**70 °C-pan**	57.45 ± 0.04 ^d^	69.66 ± 0.02 ^c^	70.71 ± 0.02 ^b^	72.07 ± 0.01 ^a^	7.55 ± 0.03 ^a^	3.85 ± 0.01 ^b^	3.03 ± 0.01 ^c^	2.25 ± 0.01 ^d^	27.30 ± 0.04 ^a^	27.22 ± 0.02 ^b^	27.04 ± 0.01 ^c^	26.61 ± 0.01 ^d^
**80 °C-pan**	57.45 ± 0.04 ^d^	69.52 ± 0.03 ^c^	72.35 ± 0.02 ^a^	71.35 ± 0.04 ^b^	7.55 ± 0.03 ^a^	4.24 ± 0.01 ^b^	1.76 ± 0.01 ^c^	1.48 ± 0.02 ^d^	27.30 ± 0.04 ^a^	26.70 ± 0.03 ^b^	26.73 ± 0.05 ^b^	24.47 ± 0.03 ^c^
**80 °C-fryer**	61.06 ± 0.02 ^d^	65.87 ± 0.05 ^c^	66.03 ± 0.04 ^b^	67.15 ± 0.09 ^a^	11.06 ± 0.03 ^a^	7.60 ± 0.02 ^b^	7.13 ± 0.03 ^c^	6.43 ± 0.04 ^d^	42.21 ± 0.04 ^a^	40.13 ± 0.05 ^b^	39.27 ± 0.10 ^c^	37.59 ± 0.04 ^d^

CW—cold water. L* (lightness, 0 = black to 100 = white) and a* (redness). Values followed by different letters in the same line for each temperature duration are significantly different (*p* < 0.05).

**Table 4:** Color parameters (L*, a*, b*) of French fries pre-treated with NaCl and citric acid solutions.

Pre-Treatment	Color Parameters/Solutions Concentration								
	L* (Mean ± sd)			a* (Mean ± sd)			b* (Mean ± sd)		
Solution	0	0.05	1	0	0.05	1	0	0.05	1
**NaCl**	60.37 ± 0.03 ^c^	74.52 ± 0.03 ^a^	74.20 ± 0.01 ^b^	7.67 ± 0.01 ^a^	1.80 ± 0.04 ^c^	1.99 ± 0.02 ^b^	26.33 ± 0.03 ^c^	30.35 ± 0.13 ^b^	30.83 ± 0.02 ^a^
**Citric acid**	60.37 ± 0.03 ^c^	74.92 ± 0.03 ^b^	76.39 ± 0.01 ^a^	7.67 ± 0.01 ^a^	1.53 ± 0.02 ^b^	1.29 ± 0.01 ^c^	26.33 ± 0.03 ^c^	29.56 ± 0.09 ^b^	30.56 ± 0.04 ^a^

L* (lightness, 0 = black to 100 = white) and a* (redness). Values followed by different letters in the same line for each solution concentration are significantly different (*p* < 0.05).

**Table 5:** Texture attributes of French fries pre-treated with water.

Texture Parameters/Pre-Treatment Time												
Pre-Treatment	Hardness, [N] (Mean ± sd)				Compression at Maximum Force, mm (Mean ± sd)				Fracturability, mm (Mean ± sd)			
**Time (min)**	**0**	**15**	**60**	**120**	**0**	**15**	**60**	**120**	**0**	**15**	**60**	**120**
**CW-pan**	0.34 ± 0.10 ^b^	0.54 ± 0.07 ^a^	0.56 ± 0.13 ^a^	0.58 ± 0.19 ^a^	2.01 ± 0.50 ^a^	1.99 ± 0.36 ^a^	1.74 ± 0.55 ^a^	1.57 ± 0.37 ^a^	3.63 ± 0.15 ^a^	3.68 ± 0.12 ^a^	3.73 ± 0.06 ^a^	3.73 ± 0.08 ^a^
**Time (min)**	**0**	**5**	**10**	**15**	**0**	**5**	**10**	**15**	**0**	**5**	**10**	**15**
**60 °C-pan**	0.98 ± 0.3 ^a^	0.75 ± 0.17 ^a,b^	0.63 ± 0.16 ^b^	0.60 ± 0.15 ^b^	2.40 ± 0.24 ^a^	2.37 ± 0.45 ^a^	2.36 ± 0.53 ^a^	2.34 ± 0.40 ^a^	3.39 ± 0.28 ^a^	3.45 ± 0.20 ^a^	3.52 ± 0.21 ^a^	3.58 ± 0.20 ^a^
**70 °C-pan**	0.98 ± 0.3 ^a^	0.96 ± 0.15 ^a^	0.73 ± 0.10 ^a^	0.71 ± 0.14 ^a^	2.40 ± 0.24 ^a^	2.30 ± 0.37 ^a^	1.95 ± 0.18 ^a,b^	1.85 ± 0.21 ^b^	3.39 ± 0.28 ^a^	3.55 ± 0.25 ^a^	3.70 ± 0.03 ^a^	3.71 ± 0.05 ^a^
**80 °C-pan**	0.98 ± 0.3 ^a^	0.93 ± 0.34 ^a^	0.78 ± 0.22 ^a^	0.75 ± 0.12 ^a^	2.40 ± 0.24 ^a^	2.00 ± 0.64 ^a^	1.98 ± 0.15 ^a^	1.97 ± 0.13 ^a^	3.39 ± 0.28 ^a^	3.59 ± 0.19 ^a^	3.61 ± 0.12 ^a^	3.63 ± 0.16 ^a^

sd—standard deviation. Values followed by different letters in the same line for each pre-treatment are significantly different (*p* < 0.05).

**Table 6:** Pearson correlation between acrylamide content and color parameters (L*, a*) and moisture content of French fries.

Pre-Treatment	L*		a*		Moisture	
	*r*	*p*-Value	*r*	*p*-Value	*r*	*p*-Value
**CW-pan**	−0.996	<0.01	0.988	<0.05	−0.866	>0.05
**CW-fryer**	−0.998	<0.01	0.992	<0.01	−0.993	<0.01
**60 °C-pan**	−0.886	>0.05	0.640	>0.05	−0.955	<0.05
**70 °C-pan**	−0.993	<0.01	0.969	<0.05	−0.924	>0.05
**80 °C-pan**	−0.993	<0.01	0.923	>0.05	−0.926	>0.05
**80 °C-fryer**	−0.965	<0.05	0.981	<0.05	−0.994	<0.05
**NaCl-pan**	−0.973	>0.05	0.997	<0.05	−0.460	>0.05
**Citric acid-pan**	−0.888	>0.05	0.986	>0.05	−0.706	>0.05

*r*—correlation coefficient. Significant differences are expressed as *p* < 0.05.

**Table 7:** Pearson correlation between acrylamide content and hardness of French fries.

Pre-Treatment	Hardness	
	r	*p*-Value
**CW-pan**	−0.925	>0.05
**60 °C-pan**	0.862	>0.05
**70 °C-pan**	0.648	>0.05
**80 °C-pan**	0.754	>0.05

## Data Availability

The data presented in this study are available in this article.
